# Open notes sounds great, but will a provider’s documentation change? An exploratory study of the effect of open notes on oncology documentation

**DOI:** 10.1093/jamiaopen/ooab051

**Published:** 2021-08-17

**Authors:** Maryam Rahimian, Jeremy L Warner, Liz Salmi, S Trent Rosenbloom, Roger B Davis, Robin M Joyce

**Affiliations:** Beth Israel Deaconess Medical Center, Harvard Medical School, Boston, Massachusetts, USA; Department of General Medicine, Vanderbilt University Medical Center, Nashville, Tennessee, USA; Beth Israel Deaconess Medical Center, Boston, Massachusetts, USA; Department of General Medicine, Vanderbilt University Medical Center, Nashville, Tennessee, USA; Beth Israel Deaconess Medical Center, Harvard Medical School, Boston, Massachusetts, USA; Beth Israel Deaconess Medical Center, Harvard Medical School, Boston, Massachusetts, USA

**Keywords:** open notes, oncology, EHR, readability, assessment and plan, 21st century cures act, information blocking

## Abstract

**Objective:**

The effects of shared clinical notes on patients, care partners, and clinicians (“open notes”) were first studied as a demonstration project in 2010. Since then, multiple studies have shown clinicians agree shared progress notes are beneficial to patients, and patients and care partners report benefits from reading notes. To determine if implementing open notes at a hematology/oncology practice changed providers’ documentation style, we assessed the length and readability of clinicians’ notes before and after open notes implementation at an academic medical center in Boston, MA, USA.

**Materials and Methods:**

We analyzed 143 888 notes from 60 hematology/oncology clinicians before and after the open notes debut at Beth Israel Deaconess Medical Center, from January 1, 2012 to September 1, 2016. We measured the providers’ (medical doctor/nurse practitioner) documentation styles by analyzing character length, the number of addenda, note entry mode (dictated vs typed), and note readability. Measurements used 5 different readability formulas and were assessed on notes written *before* and *after* the introduction of open notes on November 25, 2013.

**Results:**

After the introduction of open notes, the mean length of progress notes increased from 6174 characters to 6648 characters (*P* < .001), and the mean character length of the “assessment and plan” (A&P) increased from 1435 characters to 1597 characters (*P* < .001). The Average Grade Level Readability of progress notes decreased from 11.50 to 11.33, and overall readability improved by 0.17 (*P* = .01). There were no statistically significant changes in the length or readability of “Initial Notes” or Letters, inter-doctor communication, nor in the modality of the recording of any kind of note.

**Conclusions:**

After the implementation of open notes, progress notes and A&P sections became both longer and easier to read. This suggests clinician documenters may be responding to the perceived pressures of a transparent medical records environment.

## INTRODUCTION

Open notes is a concept that can be incorporated in institutional healthcare policies to promote patient engagement with their care by giving patients direct and complete access to their electronic health record (EHR) through the online patient portal. A pioneering multisite study conducted in 2010 enabled 20 000 patients access to their medical notes and demonstrated that shared notes improve communication between clinicians and patients and can actively engage patients in their health care.^1–7^ The growing evidence base on open notes continues to show that benefits for patients far outweigh the perceived risks that patients may be confused by their medical documentation.

As of December 2020, more than 250 organizations voluntarily adopted an open notes approach to sharing information with patients through secure, online portals, giving more than 54 million patients access to their clinical notes across North America, identifying open notes as an emerging standard of care in the United States.^8^ Beginning April 5, 2021, an “information sharing” mandate from the US government required clinicians and health systems to share all progress notes with all patients, making open notes a “law of the land.”^9–11^ Prior research indicates that patients report numerous benefits from reading their notes, such as more proactive involvement in their health care, better knowledge of their care plans, better self-care, having a deeper understanding of treatment options, and being more engaged in medical decision-making.^12–14^ Patients who read their open notes report finding inaccuracies in their records and believe reading notes helped them prepare for the informed consent process.^15^^,^^16^ Open notes clarifies communication, enabling more trusting communication between clinicians and patients.^17–21^ Patients facing cancer diagnoses have expressed considerable appreciation for open progress notes: in a recent multisite study, 98% (*n* = 3418) of cancer patients said they thought open notes was a “good idea,” over half (56%) considered reading notes helped them prepare for visits with their doctors, and very few (4%) found their notes to be confusing.^22^

Despite the national adoption of open notes in the United States, and the growing body of research indicating its safety and potential for patient engagement, open notes symbolizes a disruption in health care. While open notes is enabled by EHR platforms and online patient portals, open notes itself is *not* technology or software. Clinician concerns about real and important issues of “note bloat,” increase in the incomprehensibility of notes,^23^ clinician burnout, and the EHR have a halo effect on clinicians *before* the implementation of open notes. However, open notes as a *cause* for these burdens has not borne out. Some clinicians with note-sharing experience have reported spending more time in documentation as a result of open notes.^24^ It is unclear whether a trend toward increasing transparency and patient engagement, which was supported by recent federal mandates, will be outweighed by a trend toward verbosity and obfuscation.^25^^,^^26^

One potential challenge clinicians face when switching to open notes environment is how to document with a new audience in mind: patients, parents, and potentially care partners and proxies. In clinician surveys of open notes, most do not report changing how they document but report thinking about how they word sensitive topics related to mental health, sexual history, or obesity, and modifying language that could appear “*critical of the patient*.”^27^^,^^28^ Few investigations have looked at how documentation changes when discussing chronic debilitating illnesses, suspicion of cancer, or prognosis.^28^^,^^29^

Previously, we have shown that word-word associations and the use of standard phrases change measurably before and after open notes adoption.^28^^,^^29^ This study aims to quantify *how* clinician documentation changes, and by *how much*, in a post-open notes environment in an academic oncology healthcare setting.

## MATERIALS AND METHODS

### Study overview

The current study evaluated whether implementing open notes at a large academic medical center was associated with changes in measures of the length and readability of progress notes written by hematology/oncology clinicians.

### Subjects and setting

This study was performed at Beth Israel Deaconess Medical Center (BIDMC) in Boston, MA, USA. It included outpatient clinic notes written by Doctors of Medicine (MD) and Nurse Practitioners (NP) in the Hematology-Oncology department from January 1, 2012, to September 1, 2016. These dates bracket the November 25, 2013, open notes roll-out date. The pre-roll-out period from January 2012 to November 2013 is long enough to provide a measurable sample of the notation behavior before open notes implementation. The analysis was restricted to initial consultation notes, progress notes, and letters to other providers written by full-time MDs and NPs. NPs were included in the analysis as they take on a great role in the day-to-day care of the cancer patients and address sensitive topics such as patient distress and symptom management. Part-time faculty, fellows, and trainees were excluded from this analysis as their documentation style may have changed over time due to on-the-job training. We assumed provider-to-provider letters would have been written with a clinician end reader in mind. However, because these letters were allowed to be read by patients, we sought to explore this particular subset of notes in more detail. We included clinicians who had written a minimum of 3 letters in each of the time periods (ie, *before* and *after* open notes implementation) with the intent to observe changes attributable to each clinician. The letters were filtered to include communications between doctors; only letters beginning with the salutation “Dear Dr.” were included. The explicit bi-gram “Dear Dr.” is a template salutation that begins all clinical correspondence at BIDMC.

### Sources of the assessment and plan section of notes

The “Assessment and Plan” (A&P) is a crucial section of a progress note due to its function to outline treatment options, and as care progresses for the patient, this section remains open to revisions. Each clinician used a number of distinct annotations to indicate the A&P section. We searched for the first such expression and extracted the note beginning at these words to the beginning of the section marked *Addenda*.

### Statistical analysis

Outcomes included the following measures:

1. Length in characters of initial notes, progress notes, and letters
2. Length in characters of A&P section of the notes
3. Note type: Dictated versus Typed notation
4. Number of Addenda or Postscripts
5. Readability

Regression models compared outcomes between the periods before and after the implementation of open notes (“*before* open notes” and “*after* open notes”). We fit models using generalized estimating equations methods with a compound symmetry correlation structure to account for likely correlation among notes written by the same provider. We used an identity link function for numeric outcomes and reported the mean change between periods with the 95% confidence interval. For binary outcomes (eg, typed vs dictated), we fit log-binomial models and report risk ratios with 95% confidence intervals for the postimplementation period relative to the preimplementation period.

### Readability analysis

Readability approximates the ease with which a reader parses and comprehends a written text. Readability indices are used to assess healthcare documents.^30–32^ These scores use text attributes such as syllable counts, number of words, and number of characters to calculate an approximate reading difficulty measured by grade-level for the text. Flesch–Kincaid is the most commonly used index for general and medical documents. Simple Measure of Gobbledygook (SMOG) and Gunning Fog Index (GFI) are also recommended by Health Literacy Advisor (HLA) and the Centers for Medicaid and Medicare Services (CMS).^27^ In our study, the readability of notes was ascertained by calculating an average “grade level” using 5 common readability metrics: Flesch Kincaid, Gunning Fog Index, Coleman Liau, SMOG, and Automated Readability Index.^30–32^ A summary metric average-grade level was used in our analysis to understand that the lower the grade level, the more easy the notes should be to read. Readability scores were calculated by using the open-source R package *Readability* (https://cran.rproject.org/web/packages/readability/index.html). Numerical analyses were performed using S.A.S. (version 9.4), R (version 3.3.1), RStudio (version 0.99.903), and the R libraries *data*. *Table* and *stringr*. This study was approved by the BIDMC IRB (#2014P000158).

## RESULTS

Notes from 60 clinicians were included in this study. This included initial consultation notes from 57 clinicians, progress notes from 60 clinicians, and letters from 48 clinicians. There were 143 888 total notes and letters evaluated in the study (Table 1). Initial notes constituted 5% of all 143 888 documents analyzed. Progress Notes constituted 83%, and Letters were 12%. MDs wrote 74% of all notes; NPs wrote the remaining 26%.

**Table 1.** Number of progress notes evaluated by note type and clinician type

	Initial note, No. (%)	Progress note, No. (%)	Letter, No. (%)	Total notes
MD	6230 (4.4%)	85 125 (59.2%)	15 311 (10.6%)	106 666 (74%)
NP	855 (0.6%)	33 834 (23.5%)	2533 (1.8%)	37 222 (26%)
Totals	7085 (∼5%)	118 959 (∼83%)	17 844 (∼12%)	143 888 (100%)

### Characteristics of notes

The characteristics of notes before versus after open notes are presented in Table 2.

**Table 2.** Characteristics of notes: regression Model results^a^

Outcome	Mean before	Mean after	Mean difference (95% CI)	*P*-value
	Open notes	Open notes		
Initial note character count				
Whole section	7987	7703	−184 (−502, 133)	.26
Assessment and plan only	2279	2186	−95 (−291, 101)	.34
Number of addenda	0.51	0.40	−0.11 (−0.20, −0.01)	.03
Progress note character count				
Whole section	6174	6648	473 (177, 769)	.002
Assessment and plan only	1435	1597	161 (52, 271)	.004
Number of addenda	0.35	0.32	−0.03 (−0.07, 0.00)	.08
Letter character count	4041	4018	−23 (−256, 211)	.85

a Estimates and *P*-values from linear regression fit using generalized estimating equations methods, clustered by the provider.

#### Initial notes

There was no statistically significant change in mean character length of initial notes between the before-open notes and after-open notes periods for either the whole note (*P* = .2) or the A&P section (*P* = .34). There were fewer addenda to initial notes after the initiation of open notes (*P* = .03). The number of addenda dropped by 0.1 on average, and the 95% confidence interval is (−0.2, 0).

#### Progress notes

Progress notes were statistically significantly longer in the after-open notes period (*P* = .002 for the whole note, and *P* = .004 for the A&P section). The number of characters increased by approximately 500 overall, or by approximately 5 sentences. The A&P section increased by 160, or by about 2 sentences. Both of these increases are large compared to the note-to-note variability. The number of progress note addenda was similar in the 2 periods.

#### Letters

There were no significant changes in the length of clinician letters. Letters were about half as long as initial notes and about two-thirds as long as progress notes.

#### Modality of note entry (typed vs dictated)

There was no difference between the frequencies of typed and dictated notes between the before-open notes and after-open notes periods (Table 3). We fail to reject the null hypothesis that the use of open notes did not affect the modality of documentation.

**Table 3.** Frequency of notes by the modality of entry

Open notes	Dictated	Typed	Relative risk^a^ (95% confidence interval)	*P*-value^a^
Initial			0.92 (0.79, 1.08)	.33
Before	1222 (38%)	1981(62%)		
After	1868 (48%)	2014 (52%)		
Progress			0.90 (0.80, 1.02)	.10
Before	25 494 (51%)	24 968 (49 %)		
After	34 505 (51%)	33 992 (49 %)		

a Relative risk and *P*-values are based on log binomial models fit using generalized estimated equation methods, clustered by the provider.

### Readability of notes

Values of 5 readability metrics are broken down in Table 4.

**Table 4.** Readability of notes: regression model results^a^

Outcome	Mean before	Mean after	Mean difference (95% CI)	*P*-value
	Open notes	Open notes		
Initial notes				
Average grade level	11.72	11.54	−0.19 (−0.63, 0.27)	.42
ARI	9.67	9.46	−0.21 (−0.73, 0.31)	.43
SMOG	12.71	12.45	−0.26 (−0.66, 0.14)	.21
Coleman Liau	12.62	12.71	0.09 (−0.17, 0.36)	.50
Gunning Fog	13.80	13.50	−0.30 (−0.87, 0.27)	.30
Flesch Kincaid	9.81	9.56	−0.25 (−0.77, 0.27)	.34
Progress notes				
Average grade level	11.50	11.33	−0.17 (−0.30, −0.04)	.01
ARI	9.64	9.36	−0.29 (−0.47, −0.10)	.002
SMOG	12.26	12.05	−0.21 (−0.33, −0.09)	.0008
Coleman Liau	12.88	12.95	0.08 (−0.06, 0.21)	.25
Gunning Fog	13.34	13.13	−0.21 (−0.35, −0.07)	.004
Flesch Kincaid	9.38	9.17	−0.21 (−0.35, −0.08)	.002

a Estimates and *P*-values from linear regression fit using generalized estimating equations methods, clustered by provider.

The average of the 5 readability metrics represent a summary metric (Average Grade Level). The average readability score of the progress notes decreased (*P* value .01). Four of the 5 metrics showed significant reduction in progress note complexity and increase in readability. The 5th metric (Coleman Liau) showed a modest and statistically insignificant increase in complexity. The same 4 metrics showed a statistically insignificant drop in Initial Note complexity.

## DISCUSSION

This study was designed to better understand if an open notes setting changes how hematology-oncology clinicians document patient interactions. We focused on the basic metrics (ie, note length, A&P, readability, modality of note entry [dictated vs typed], and the number of addenda). Metrics were chosen because they were available in all notes and were directly comparable, thereby enabling quantitative analysis.

Our research uncovered a statistically significant difference in the mean character length of the complete progress notes between *before*-open notes and *after*-open notes periods. This included a statistically significant difference in mean character length of the A&P section of progress notes. Additionally, average of 5 readability metrics for all notes (Average Grade Level), decreased significantly which indicates notes became more readable.

We also observed that the character length of the A&P section increased. The A&P is where clinicians express changes in patients’ health status over time and was expected to show the most variability from note to note. The entire length of the progress notes also increased.

The increase in readability and note length *after* open notes may indicate providers began documenting in a way they believed would be more readable or easier to understand by a more diverse audience (ie, patients). Changes in increased readability scores were not reflected in the writing of initial notes, which remained consistent in length and written at a higher grade level. This may demonstrate open notes prompt clinicians to avoid jargon and acronyms in sections of notes (eg, A&P) they believe to be most used by patient readers. This kind of transparency creates an opportunity to foster a greater sense of coordination between clinicians and patients. Our primary focus on the A&P removed the “review of systems” and “medication history” sections of notes, both of which are commonly templated or auto-populated from other areas of the medical record at BIDMC. As such, results presented are an undiluted view of efforts made by oncologists to communicate more clearly about treatment options and goals with their patients. It is unknown whether these efforts were made by conscious decision by the oncologists in response to open notes. A future qualitative interview with the clinicians who wrote these notes may better reveal the decision-making process that went into the changes made over time.

The drop in complexity of progress notes and not of initial notes may signal that initial notes were not affected by the open notes environment. However, a close inspection of the modest trend in initial note complexity is consistent with the result from progress notes, so perhaps the drop in initial note complexity is more noteworthy than at first glance. A Bayesian analysis in which each group acts as a prior for the other may allow us to conclude that this drop was not coincidental, and so is statistically significant conditional on the progress notes acting as a prior. The statistical significance does not imply clinical significance, but the direction of change, however small the effect, is consistent with the hypothesis that open notes does encourage and enable patient communication by enhancing clarity and readability, and reducing overall complexity.

Finally, we uncovered a downward shift in the number of addenda to initial notes between *before*-open notes and *after*-open notes periods. This finding may allay clinicians’ concerns that open notes may cause a substantial increase in addenda to address feedback from patients. It is also notable that a preformatted text as an addendum was added to all notes that exceeded a preset duration of note signature delay. This addendum was applied prior to the signature of the writer as an attestation of authenticity (Supplementary Appendix). The decrease in number of addenda of initial notes is consistent with our previous findings that open notes environment encouraged clinicians to finish notes on time, and sign notes earlier than *before*-open notes.^28^

We had several limitations in our research that demanded a detailed discussion. Language-based analysis is limited by the degree to which words or phrases may be reduced to quantitative metrics. In this study, we used 2 simple metrics for the counting of note length and number of addenda, however, other metrics might be applicable. For example, many clinicians used multiple equivalent terms in the same note which may have benefitted from a synonymy analysis. Moreover, to know if it took clinicians more time to choose alternative words and phrases in an effort to communicate with a patient reader (eg, *hospice* or *terminal)* we could have looked at the connotations, words chosen, and the use of euphemisms. There are worries clinicians who make overtures to write a note for patient readers might lose the objective recitation of facts and invalidate the clinical utility of the note.^33^ This is an area in need of additional linguistic research. The assumption that note length and number of addenda correlated with information is clearly only approximate. The mode of communication (dictated vs typed) is a behavioral measure of clinician preference, which does not attempt to quantify communication. Lastly, readability metrics focus on rudimentary word and sentence choice. They do not seek to quantify concept or content as simple prose can be “read” by. For instance, a reader may be at the 8th grade Lexile level, but the conceptual content might be far above the cognitive level of that 8th grader. Taken together these metrics describe the general phenomena but not in a granular or finely detailed manner of the notes, but they cannot ascertain the intention of clinician to convey or withhold information. This is significant because another study that might use a more finely detailed linguistic “capture” might prove details that can be more intensely examined as per word choice, inflection, or subtext as discussed in this section.

The methods described here may help identify changes in oncology clinician documentation style in an open notes environment. Using these metrics, we were able to determine the A&P portion of an oncology note became both longer and easier to read over the time period studied, which added to the overall word count in a note.

## CONCLUSION

Our methods quantify how clinician documentation changes, and by how much, in a post-open notes environment in an academic oncology healthcare setting. That there is a noticeable change is consistent with the preconception that clinicians will change their documentation when patient readers are taken into consideration in an effort to communicate more clearly with patients—in both detail and with more simple and transparent language. It is unknown whether the clinicians whose notes were studied were intentional in their change in documentation style; however, the results of A&P expansion and tendency to add more words align with the preferences of patients who read their notes. A recent multisite study of patients who read their visit notes showed 96% (*n* = 20 813) reported understanding all or nearly all of their note.^34^ However even patients who understand their notes said clinicians could make notes more meaningful by “*restructuring notes to put the more meaningful information at the top*,” and avoiding medical jargon and spelling out acronyms. This analysis shows documentation does change after open notes, but it is unknown whether this was a conscious choice on behalf of the clinicians studied. A qualitative interview with open note writers is a logical next step to better understanding this emerging environment.

## FUNDING

This work was conducted with support from Harvard Catalyst, Harvard Clinical and Translational Science Center, National Center for Research Resources, National Center for Advancing Translational Sciences, National Institutes of Health Award (UL1 TR002541) and financial contributions from Harvard University and its affiliated academic healthcare centers.

## AUTHOR CONTRIBUTIONS

Data extraction and analysis: MR, RBD, JLW, RMJ. Data interpretation: all authors. Manuscript writing: all authors. Manuscript revision: all authors. Final approval of the manuscript: all authors.

## SUPPLEMENTARY MATERIAL

Supplementary material is available at *Journal of the American Medical Informatics Association* online.

## CONFLICT OF INTEREST STATEMENT

JLW: stock and other ownership interests—HemOnc.org. RMJyce: stock and other ownership interests—Intellipharmaceutics. No other potential conflicts of interest.

## DATA AVAILABILITY

The raw patient data relevant to this study cannot be shared; it would compromise patient privacy and violate institutional policies and legal requirements.

## Supplementary Material

ooab051_Supplementary_Data
