# Supplementary material for: Open notes sounds great, but will a provider’s documentation change? An exploratory study of the effect of open notes on oncology documentation
Source: JAMIA Open. 2021 Aug 17;4(3):ooab051. doi: 10.1093/jamiaopen/ooab051 (PMC8518311; doi:10.1093/jamiaopen/ooab051)
Supplement: ooab051_Supplementary_Data [file ooab051_supplementary_data.pdf]

Appendix:

I, _________, attest that the medical record entry for ________ accurately reflects the documentation I made when I assessed, diagnosed, treated, and/or communicated about the above named patient. I also attest that this information is true, accurate, and complete to the best of my knowledge and I understand that any falsification, omission, or concealment of material fact may subject me to administrative, civil, or criminal liability.
